# *Plasmodium* sporozoites on the move: Switching from cell traversal to productive invasion of hepatocytes

**DOI:** 10.1111/mmi.14645

**Published:** 2020-12-05

**Authors:** Manon Loubens, Laetitia Vincensini, Priyanka Fernandes, Sylvie Briquet, Carine Marinach, Olivier Silvie

**Affiliations:** ^1^ Centre d’Immunologie et des Maladies Infectieuses Sorbonne Université, INSERM, CNRS, CIMI‐Paris Paris France

**Keywords:** apical organelles, cell traversal, host cell invasion, malaria, *Plasmodium sporozoites*

## Abstract

Parasites of the genus *Plasmodium*, the etiological agent of malaria, are transmitted through the bite of anopheline mosquitoes, which deposit sporozoites into the host skin. Sporozoites migrate through the dermis, enter the bloodstream, and rapidly traffic to the liver. They cross the liver sinusoidal barrier and traverse several hepatocytes before switching to productive invasion of a final one for replication inside a parasitophorous vacuole. Cell traversal and productive invasion are functionally independent processes that require proteins secreted from specialized secretory organelles known as micronemes. In this review, we summarize the current understanding of how sporozoites traverse through cells and productively invade hepatocytes, and discuss the role of environmental sensing in switching from a migratory to an invasive state. We propose that timely controlled secretion of distinct microneme subsets could play a key role in successful migration and infection of hepatocytes. A better understanding of these essential biological features of the *Plasmodium* sporozoite may contribute to the development of new strategies to fight against the very first and asymptomatic stage of malaria.

## Graphical abstract

*Plasmodium* sporozoites, the mosquito‐transmitted forms of the malaria parasite, traverse multiple cells during their migration from the skin to the liver, before switching to productive invasion of a suitable hepatocyte for replication. Cell traversal and productive invasion are functionally independent processes that require proteins secreted from specialized secretory organelles known as micronemes. This review summarizes what is currently known of the sporozoite invasion modes, and discusses the role of microneme heterogeneity and environmental sensing in switching from a migratory to an invasive state.

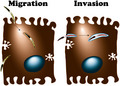

## 1 INTRODUCTION

Malaria is caused by *Plasmodium* spp., a protozoan of the phylum of Apicomplexa that is transmitted to mammalian hosts by female *Anopheles* mosquitoes. Infection begins with the inoculation of motile sporozoites into the host dermis by infected mosquitoes. Some of these sporozoites enter the bloodstream and traffic to the liver, where they actively invade hepatocytes to differentiate into thousands of merozoites (Frischknecht & Matuschewski, 2017). Once released into the blood, merozoites invade and multiply inside erythrocytes, causing the symptoms and complications associated with malaria. Infection of the liver is an essential and clinically silent phase of the parasite life cycle, and has long been considered as an ideal target for a malaria vaccine (Duffy & Patrick Gorres, 2020), since blocking parasite progression from the liver to the blood not only protects against the pathology, but also abolishes parasite transmission to the mosquito vector.

Invasive stages of *Plasmodium* and other apicomplexan parasites are polarized cells characterized by specialized apical secretory organelles termed micronemes and rhoptries whose regulated secretion is required for active migration and subsequently for host cell invasion. The function of these organelles and the signaling pathways involved in their secretion have been extensively studied, especially in *Plasmodium* blood‐stage merozoites and its sister apicomplexan *Toxoplasma* tachyzoites (reviewed in Dubois & Soldati‐Favre, 2019). However, in contrary to these stages that rapidly invade cells, sporozoites migrate extensively before ultimately committing to productive host cell invasion, at the right time and in the right place, within the liver parenchyma.

**FIGURE 1:**
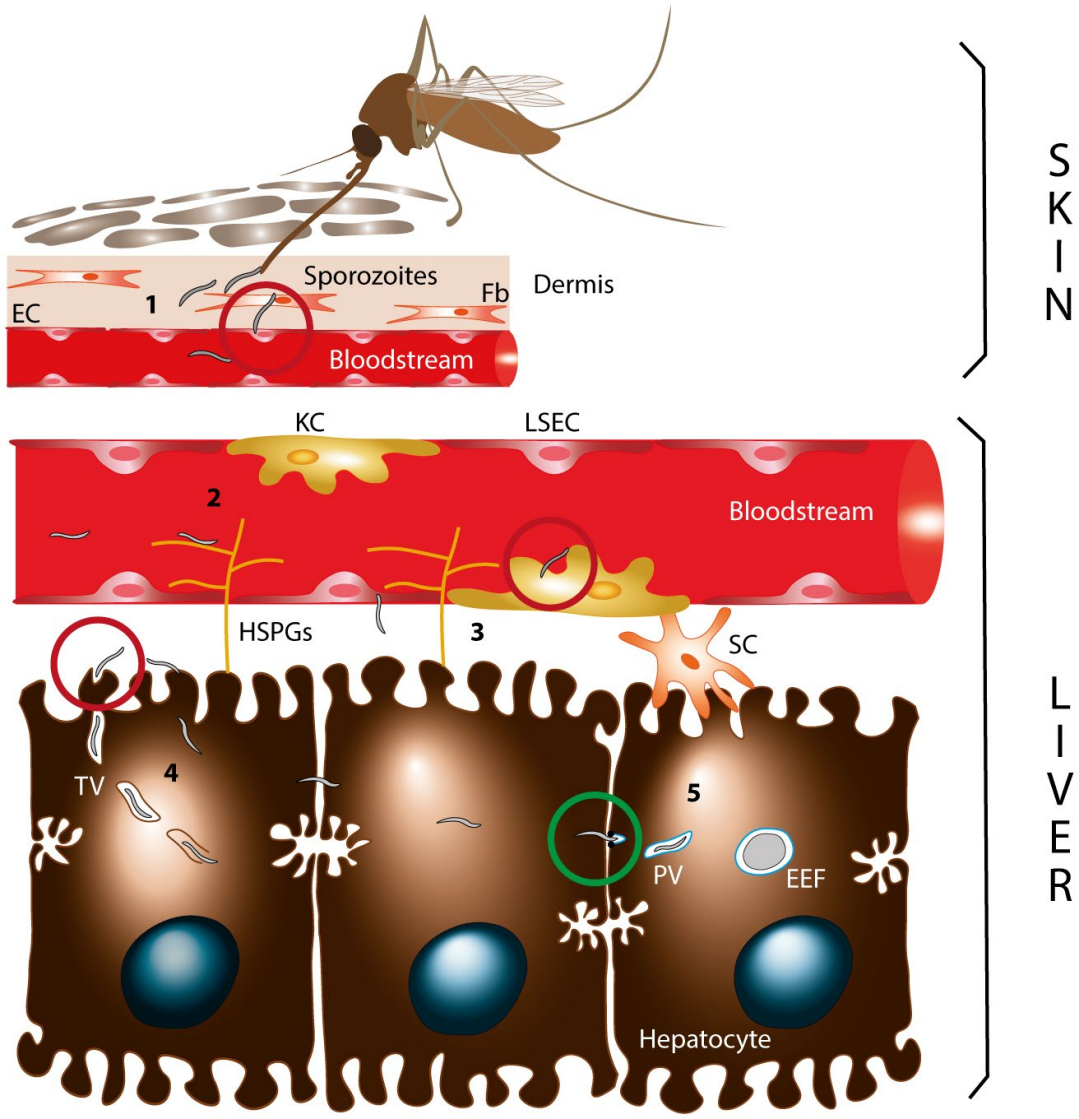
The *Plasmodium* sporozoite journey from the skin to the liver. *Plasmodium* sporozoites are deposited in the dermis of the mammalian host by the bite of an infected anopheline mosquito (1). They cross dermal fibroblasts (Fb) and endothelial cells (EC), enter the blood circulation and traffic to the liver. Sporozoites are sequestered in the liver sinusoids through interactions between the circumsporozoite protein (CSP) and hepatic heparan sulfate proteoglycans (HSPGs) (2), then, cross the sinusoidal cellular barrier through liver sinusoidal endothelial cells (LSECs) or Kupffer cells (KCs) (3). In the liver parenchyma, sporozoites traverse through several hepatocytes, sometimes inside a transient vacuole (TV) (4), before switching to productive invasion of a final hepatocyte with formation of the parasitophorous vacuole (PV) (5). Inside the PV, the parasite develops into a replicative exoerythrocytic form (EEF). Sporozoite cell traversal and productive invasion events are indicated by red and green circles, respectively

Sporozoite migration from the inoculation site in the skin to their final destination in the liver requires crossing through several cellular barriers (Figure 1), including dermal fibroblasts, endothelial cells, liver sinusoidal endothelial cells (LSECs) and Kupffer cells (KCs) which are the liver resident macrophages (Amino et al., 2008; Ishino et al., 2004; Tavares et al., 2013). While KCs were long thought to act as an essential portal for sporozoites in the liver (Frevert et al., 2008; Pradel & Frevert, 2001), intravital imaging revealed that sporozoites can migrate through either KCs or LSECs or use a paracellular route to reach the underlying hepatocytes (Tavares et al., 2013). Nevertheless, mutants that are deficient for cell traversal (CT) fail to leave the skin when injected into the dermis (Amino et al., 2008), and cannot cross the liver sinusoidal barrier when directly injected into the blood circulation (Ishino et al., 2005a; Ishino et al., 2004; Yang, O’Neill, et al., 2017), thus, establishing a crucial role for CT in vivo, for sporozoite migration through tissues. Additionally, sporozoites may also use CT to escape phagocytosis by KCs (Tavares et al., 2013). During CT, sporozoites enter, pass through, and exit from host cells (Mota et al., 2001). Sporozoites can either breach the cell plasma membrane upon entry, or first form transient vacuoles (TVs) from which they egress by rupturing the vacuole membrane before exiting the cell (Risco‐Castillo et al., 2015) (Figure 1). It is possible that a similar process may operate in the mosquito during sporozoite invasion of salivary glands (Ando et al., 1999; Pimenta et al., 1994; Sinden & Strong, 1978).

In the liver parenchyma, *Plasmodium* sporozoites traverse several hepatocytes before switching to productive invasion of a final one (Frevert et al., 2005; Mota et al., 2001). Productive invasion is characterized by the formation of a specialized membrane‐bound compartment, the parasitophorous vacuole (PV), within which the parasite replicates into thousands of erythrocyte‐invasive merozoites (Frischknecht & Matuschewski, 2017) (Figure 1). By analogy with other apicomplexan invasive stages, sporozoites presumably form their PV by invading cells through a so‐called moving junction (MJ) (reviewed in Cowman et al., 2017; Frénal et al., 2017). The MJ is assembled at a site of close apposition of the host cell and the parasite membranes, and resolves as a ring through which the parasite actively penetrates inside an invagination of the host cell plasma membrane, leading to the formation of the PV (Riglar et al., 2011; Shen & Sibley, 2012). Importantly, despite indirect evidence suggesting that *Plasmodium* sporozoites enter cells through a MJ (Ishino et al., 2019; Risco‐Castillo et al., 2015; Risco‐Castillo et al., 2014; Silvie et al., 2004; Yang, Lopaticki, et al., 2017), such events have proved difficult to capture by imaging approaches, and thus, the molecular nature of the sporozoite MJ remains elusive (Bargieri et al., 2014). During parasite internalization, the PV membrane (PVM) is remodeled, with selective exclusion of some host membrane proteins, resulting in the formation of a nonfusogenic compartment within which the parasite safely develops into liver stages (Risco‐Castillo et al., 2015). The PVM is further remodeled postinvasion with the integration of parasite‐derived proteins, including UIS4, an abundant PVM protein that plays a major role in liver stage development (Mueller et al., 2005) and has been widely used as a liver stage PVM marker.

## 2 CHALLENGES IN STUDYING *PLASMODIUM* SPOROZOITE INVASION

The biology of sporozoites has been mainly explored in the rodent malaria parasites *P. berghei* and *P. yoelii*, since the entire life cycle of these species can be reproduced in the laboratory through cycling between mice and mosquitoes. In addition, *P. berghei* and *P. yoelii* are readily amenable to genetic modification (Janse et al., 2006). Diverse transgenic lines expressing fluorescent markers or harboring targeted gene disruptions have been instrumental in the characterization of parasite factors mediating motility, CT or host cell infection (Janse et al., 2011). Fewer studies have investigated the mechanisms of sporozoite infection in *P. falciparum*, the main species infecting humans, notably due to the challenge of producing high quality gametocytes in vitro and the need for permissive cell culture systems such as primary human hepatocytes (Mazier et al., 1985). Recent progress in genome editing based on the CRISPR‐Cas9 technology has facilitated the production of genetically modified *P. falciparum* strains for functional studies in sporozoites (Miyazaki et al., 2020; Tibúrcio et al., 2019; Yang, Lopaticki, et al., 2017). In contrast, similar molecular genetics approaches cannot be applied to *P. vivax,* the second most important human malaria parasite, due to the absence of a blood‐stage culture system. This also restricts access to *P. vivax* gametocytes, which can only be obtained from the blood of infected individuals for experimental transmission to mosquitoes, thereby limiting studies on sporozoites from this species.

Sporozoite invasion encompasses both CT, sometimes within TVs, and productive invasion, with the formation of a PV. A number of assays have been developed to quantify CT activity, including a cell wound‐repair assay based on the uptake of fluorescence dextran by traversed cells (Mota et al., 2001; Sinnis et al., 2012). Alternative assays rely on the release of intracellular dyes such as calcein green by wounded cells (Sinnis et al., 2007), or the uptake of propidium iodide, which allows the visualization of CT by live imaging, both in vitro and in vivo (Formaglio et al., 2014).

The fact that sporozoites can form replicative vacuoles (PVs) or transient nonreplicative vacuoles (TVs) illustrates the difficulty in disentangling both invasion modes (Risco‐Castillo et al., 2015). Moreover, productive invasion is a rare event that is difficult to capture among CT events, and less than 20% of salivary gland sporozoites are infective, at least in vitro (Risco‐Castillo et al., 2015). Several methods that were developed to quantify sporozoite invasion rely on counting intracellular parasites. Transgenic sporozoites expressing fluorescent proteins enable a direct quantification of sporozoite‐containing cells by flow cytometry (Prudêncio et al., 2008). Alternatively, intracellular parasites can be visualized by immunofluorescence assays based on differential labeling of extra‐ versus intracellular sporozoites (Renia et al., 1988; Sinnis et al., 2012). These methods do not distinguish between sporozoites in the process of traversal from those that have productively invaded. In nonpermissive cells, intracellular sporozoites only correspond to traversing parasites, while in permissive cells both traversing and productively invaded sporozoites inside a PV can be observed. Comparison of sporozoite invasion rates in permissive and nonpermissive cell lines thus provides an indirect mean to assess productive invasion (Risco‐Castillo et al., 2015; Silvie et al., 2006). It should be noted that CT‐deficient mutant sporozoites can accumulate inside TVs, resulting in an apparent increase in total invasion events, although productive invasion is not affected (Risco‐Castillo et al., 2015; Steel et al., 2018), thus, making it difficult to distinguish between both invasion modes. Of note, the formation of TVs has been documented with *P. yoelii* and *P. berghei* sporozoites, in both hepatic and nonhepatic cells (Bindschedler et al., 2020; Risco‐Castillo et al., 2015), but has not been reported so far with *P. falciparum*. While the presence of a membrane surrounding intracellular sporozoites is insufficient to discriminate between TVs and PVs, the exclusion of host membrane proteins from the vacuole membrane is a distinctive feature of PVs (Risco‐Castillo et al., 2015). Productive invasion is also associated with a discharge from the sporozoite rhoptry organelles, which provides another indirect marker for productive invasion (Risco‐Castillo et al., 2015; Risco‐Castillo et al., 2014). Finally, a simple way to assess productive invasion is by counting exoerythrocytic forms (EEFs), the replicative forms resulting from parasite development inside the PV (Sinnis et al., 2012). However, while normal numbers of EEFs are indicative of the absence of any invasion defect, reduced EEF numbers can result from numerous factors such as reduced invasion, defects in parasite intracellular survival or development.

## 3 CELL TRAVERSAL AND PRODUCTIVE INVASION ARE FUNCTIONALLY INDEPENDENT PROCESSES

*P. yoelii* sporozoites productively invade host cells after a 30–60 min delay in vitro, coinciding with the end of CT activity (Risco‐Castillo et al., 2015), which suggests that CT and productive invasion are temporally controlled events in this species. Similar observations have been made with *P. berghei* sporozoites (Manzoni et al., 2017). Importantly, CT and productive invasion depend on distinct parasite effectors, suggesting that they may be mechanistically independent processes. Several parasite proteins have been identified through reverse genetic studies as essential mediators of CT, including the Sporozoite Protein Essential for Cell Traversal (SPECT)‐1, the perforin‐like protein (PLP)‐1 (also called SPECT‐2), and the Cell Traversal protein for Ookinetes and Sporozoites (CelTOS) (Ishino et al., 2005a; Ishino et al., 2004; Kariu et al., 2006). PLP‐1 belongs to an evolutionarily conserved family of pore‐forming proteins (Kaiser et al., 2004), and recombinant *P. falciparum* PLP‐1 was shown to have membrane lytic activity (Garg et al., 2013), consistent with a central role of the protein in rupturing host cell membranes during CT. PLP1‐deficient sporozoites can still enter cells inside TVs but fail to egress (Risco‐Castillo et al., 2015). Pores formed by PLP‐1 probably contribute to host cell membrane destabilization, but could also mediate the trafficking of other parasite effectors. In contrast, the molecular function of SPECT‐1 and CelTOS is currently unknown. CelTOS was shown to bind phosphatidic acid, a lipid present within the inner leaflet of plasma membranes, suggesting that CelTOS could be involved in membrane breaching when sporozoites exit from traversed cells (Jimah et al., 2016). A more recent study confirmed the role of CelTOS during CT in *P. yoelii* and uncovered an additional role of the protein in sporozoite gliding motility (Steel et al., 2018). Remarkably, mutant sporozoites lacking either SPECT‐1, PLP‐1 or CelTOS fail to traverse through cells and show defects in parasite migration both in the skin and the liver, but still retain a normal capacity to productively invade hepatocytes in vitro (Ishino et al., 2005a; Ishino et al., 2004; Kariu et al., 2006; Risco‐Castillo et al., 2015; Steel et al., 2018; Yang, O’Neill, et al., 2017), indicating that the main physiological role of CT is to cross cellular barriers and that the CT machinery is not required for subsequent productive invasion.

*Plasmodium* sporozoites can traverse diverse cell types in vitro (Coppi et al., 2007; Mota et al., 2001; Yang, O’Neill, et al., 2017), questioning the role of tissue‐specific host receptors during cell traversal. However, CT efficiency may vary depending on the type of cells. In this regard, a systematic extracellular protein screening approach identified the host integrin αvβ3 as a host receptor for *P. falciparum* thrombospondin‐related anonymous protein (TRAP) (Dundas et al., 2018). TRAP plays a critical role in motility (Sultan et al., 1997), and was shown to be secreted onto the sporozoite surface and shed during invasion (Ejigiri et al., 2012; Silvie et al., 2004). TRAP harbors two adhesive modules in its ectodomain, an integrin‐like von Willebrand factor A domain (I domain) and a thrombospondin type I repeat (TSR domain) (Matuschewski et al., 2002), and is connected to the parasite internal motor machinery via its cytoplasmic tail. Dundas et al proposed that interactions between TRAP and integrin αvβ3 could play a role during sporozoite migration in the dermis (Dundas et al., 2018). Another study recently showed that the integrin‐like I domain of TRAP can be replaced by evolutionary distant I domains, suggesting that TRAP, while not involved in host cell tropism, may act as a poly‐specific ligand allowing sporozoite migration through diverse tissues (Klug et al., 2020). In the liver, CD68 was identified as a Kupffer cell surface protein required for sporozoite internalization (Cha et al., 2015), and was shown to act by binding to the parasite glyceraldehyde 3‐phosphate dehydrogenase (GAPDH) exposed on the surface of sporozoites (Cha et al., 2016), yet, the function of this interaction remains unknown.

The molecular mechanisms underlying sporozoite productive invasion of hepatocytes also remain poorly understood. Two members of the 6‐cysteine (6‐cys) domain protein family (Arredondo & Kappe, 2017), P36 and P52, play an essential role in sporozoite infection across various *Plasmodium* species (Arredondo et al., 2018; Ishino et al., 2005b; Labaied et al., 2007; Manzoni et al., 2017; van Schaijk et al., 2008). Both rodent and *P. falciparum* sporozoites deficient for P36 and/or P52 fail to invade and form a PV in hepatocytes, but do not show any defect in CT activity (Arredondo et al., 2018; Ishino et al., 2005b; Labaied et al., 2007; Manzoni et al., 2017; van Schaijk et al., 2008). In addition, two host membrane proteins, the tetraspanin CD81 and the Scavenger Receptor Class B Type 1 (SR‐B1), are also necessary for sporozoite productive invasion (Foquet et al., 2014; Manzoni et al., 2017; Rodrigues et al., 2008; Silvie et al., 2003; Yalaoui et al., 2008). CD81 and SR‐B1 define independent entry routes for both human and rodent parasites (Manzoni et al., 2017). *P. yoelii* sporozoites strictly depend on CD81 (but not SR‐B1) to infect cells (Manzoni et al., 2017; Silvie et al., 2003), and antibodies against CD81 (but not SR‐B1) inhibit invasion of primary human hepatocytes by *P. falciparum* sporozoites (Dumoulin et al., 2015; Foquet et al., 2014; Manzoni et al., 2017; Silvie et al., 2003). Of note, immortalized hepatocyte cell lines poorly support *P. falciparum* sporozoite infection, even when CD81 is over‐expressed, indicating that other factors in addition to CD81 are required for productive invasion in this species (Dumoulin et al., 2015; Sattabongkot et al., 2006; Silvie et al., 2006; Tweedell et al., 2019). In contrast to *P. yoelii*, *P. berghei* sporozoites can use SR‐B1 as an alternative entry pathway in the absence of CD81 (Manzoni et al., 2017), at least in human cells (Langlois et al., 2020). Both CD81 and SR‐B1 individually support *P. berghei* productive invasion of host cells, and function independently one from the other (Manzoni et al., 2017). Importantly, CD81 and SR‐B1 are not required for sporozoite CT (Manzoni et al., 2017; Silvie et al., 2006; Silvie et al., 2003). In the absence of host CD81, there is no rhoptry discharge in *P. yoelii* sporozoites (Risco‐Castillo et al., 2014). CD81 may thus act at an early step of the invasion process, upstream of MJ formation, possibly in sporozoite attachment and/or activation for rhoptry secretion. Inter‐species genetic complementation has revealed that transgenic *P. berghei* sporozoites expressing *P. yoelii* P36 infect cells using CD81 but not SR‐B1, similar to *P. yoelii* sporozoites, while reciprocal expression of *P. berghei* P36 in transgenic *P. yoelii* renders sporozoites capable of using an alternative SR‐B1‐dependent entry route (Manzoni et al., 2017). This functional link between P36 and host cell receptors raises the possibility that P36 interacts with CD81, SR‐BI, or associated host cell partners, committing the parasite to host cell entry. In another study, the host EphA2 protein was proposed as a crucial entry factor for sporozoites, acting as a receptor for P36 (Kaushansky et al., 2015). However, the contribution of EphA2, if any, is not clear as sporozoites can invade cells independently of EphA2 (Langlois et al., 2018).

Studies of host cell invasion by *Plasmodium* merozoites or *Toxoplasma* tachyzoites have shown that formation of the MJ involves the export of a complex of rhoptry neck (RON) proteins, including RON2, RON4, and RON5, into the host cell. RON2 is inserted into the host cell membrane and acts as a receptor for the protein Apical Membrane Antigen 1 (AMA1), which is secreted from the micronemes onto the parasite surface (reviewed in Besteiro et al., 2011; Cowman et al., 2017; Frénal et al., 2017). Both AMA1 and RONs are expressed in sporozoites (Silvie et al., 2004; Srinivasan et al., 2004; Tokunaga et al., 2019; Tufet‐Bayona et al., 2009). In *P. berghei*, RON proteins play an important role during invasion of mosquito salivary glands and mammalian hepatocytes (Giovannini et al., 2011; Ishino et al., 2019; Nozaki et al., 2020), while the role of AMA1 in *Plasmodium* sporozoites is still unclear (Bargieri et al., 2014). While anti‐AMA1 antibodies were shown to inhibit *P. falciparum* sporozoite infection in human hepatocytes (Silvie et al., 2004), a stage‐specific gene knockout revealed that AMA1 is dispensable for sporozoite CT and productive invasion in *P. berghei* (Giovannini et al., 2011). It is possible that alternative sporozoite ligands may compensate for the absence of AMA1, as observed in *T. gondii* (Lamarque et al., 2014). One such candidate is the Merozoite Apical Erythrocyte‐Binding Ligand (MAEBL), a protein that contains two AMA1‐like domains in its extracellular domain, and plays an important role for invasion of the mosquito salivary gland (Kariu et al., 2002; Saenz et al., 2008). Recently, Yang et al reported that AMA1 and MAEBL both play important roles during sporozoite cell traversal and liver infection in *P. falciparum* (Yang, Lopaticki, et al., 2017), consistent with previous reports that anti‐MAEBL antibodies inhibit sporozoite infectivity (Peng et al., 2016; Preiser et al., 2004). Whether CT is a specific function of *P. falciparum* AMA1 and MAEBL or whether these proteins play a conserved role in sporozoite adhesion rather than invasion, as proposed for AMA1 (Bargieri et al., 2013), requires further investigation.

## 4 THE ROLE OF ENVIRONMENTAL SENSING IN THE SWITCH BETWEEN SPOROZOITE TRAVERSAL AND INVASION MODES

The molecular mechanisms underlying the switch in *Plasmodium* sporozoites from a migratory to an invasive mode are still unclear. CT itself has been proposed to activate sporozoites for commitment to productive invasion, possibly via exposure to cytoplasmic components (Mota et al., 2002). In line with this idea, exposure of sporozoites to high potassium (K^+^) concentration, mimicking the intracellular environment, was shown to decrease CT activity and enhance productive invasion (Kumar et al., 2007). However, it has now been established that CT‐deficient sporozoites infect hepatocytes with normal efficiency in vitro (Ishino et al., 2005a; Ishino et al., 2004; Kariu et al., 2006), and with similar kinetics as WT parasites (Risco‐Castillo et al., 2015), thereby excluding the need for prior contact with the host cell cytoplasm for parasite activation.

Two competing models have been proposed to explain how sporozoites switch from migration to invasion (Frischknecht & Matuschewski, 2017). The first model suggests that the parasite requires activating signals to commit to productive invasion (switch‐on model) (Coppi et al., 2007; Ishino et al., 2005b; Mota et al., 2002), whereas the second model postulates that sporozoites productively invade upon inactivation of CT factors (switch‐off model) (Amino et al., 2008). The latter was based on the observation that CT‐deficient mutant sporozoites invade cells more rapidly and efficiently than WT parasites. However, this apparent increase in invasion events was later shown to result from the retention of CT‐deficient sporozoites inside non replicative TVs (Risco‐Castillo et al., 2015). The observation that sporozoites become competent for productive invasion only after a delay, even when CT is abrogated, is more consistent with a switch‐on model (Risco‐Castillo et al., 2015). In both models, environmental stimuli and/or intrinsic factors may contribute to the commitment to productive invasion.

*P. berghei* sporozoites can invade and develop inside diverse cell types in vitro, yet, selectively infect hepatocytes in rodent hosts (Tavares et al., 2017), suggesting that sporozoite activation is triggered in the liver. Liver heparan sulfate proteoglycans (HSPGs) have been identified as central regulators of *Plasmodium* sporozoite behavior, promoting the switch from a migratory to an invasive state (Coppi et al., 2007). Sporozoites bind to the glycosaminoglycan chains (GAGs) of hepatocyte HSPGs, which protrude from the space of Disse into the liver sinusoids, and are among the most highly sulfated proteoglycans in the mammalian host (Pradel et al., 2002; Sinnis & Coppi, 2007). A high sulfation density of HSPGs was shown to be required for sporozoite binding (Pinzon‐Ortiz et al., 2001). *P. berghei* sporozoites migrate through cells expressing low‐sulfated HSPGs, such as those in the skin, while highly sulfated HSPGs of hepatocytes activate sporozoites for invasion (Coppi et al., 2007). HSPGs bind to the circumsporozoite protein (CSP), an interaction that contributes to sporozoite sequestration in the liver sinusoids (Frevert et al., 1993). CSP is the most abundant protein on the sporozoite surface where it forms a dense coat and plays multiple roles in sporozoite development, gliding motility, and interactions with host cells. It consists of a highly conserved N‐terminal charged region named Region I, variable central repeats, a C‐terminal region harboring a TSR domain and a GPI anchor for insertion into the sporozoite plasma membrane. CSP is proteolytically cleaved upon contact with the liver HSPGs, a process that contributes to activation of sporozoites (Coppi et al., 2007). In fact, in *P. berghei* sporozoites, inhibition of CSP cleavage by the cysteine protease inhibitor E64, or by deletion of CSP region I, is associated with decreased CT activity and enhanced invasion (Coppi et al., 2011; Coppi et al., 2005). Cleavage of *P. berghei* CSP is associated with conformational changes in the protein and exposure of the C‐terminal TSR domain, which upon recognition of host factors may participate in signal transduction and commitment to infection. Another study showed that *P. falciparum* CSP also undergoes N‐terminal processing in the mosquito, associated with a reversible conformational change masking epitopes in the N‐ and C‐terminal regions of the protein until sporozoites interact with hepatocytes (Herrera et al., 2015). Importantly, sporozoites continue to migrate through cells even after contact with hepatocytes, especially in vitro, indicating that CSP cleavage promoted by contact with highly sulfated HSPGs in the liver is an upstream event of a complex activation process. CSP is considered as a major target for vaccine development, and CSP antibodies can confer protection through various effector mechanisms, including inhibition of sporozoite motility (Flores‐Garcia et al., 2018; Vanderberg & Frevert, 2004), inhibition of protein cleavage (Espinosa et al., 2015; Kisalu et al., 2018), and parasite killing (Aliprandini et al., 2018).

In addition to HSPGs, CD81 and SR‐B1 are important host factors for sporozoite productive invasion, yet, their molecular function remains unknown. In the presence of CD81‐expressing cells, *P. yoelii* sporozoites become competent for productive invasion only after a delay (>30 min), independently of cell traversal activity (Risco‐Castillo et al., 2015). Similar results were also obtained with *P. berghei* sporozoites (Manzoni et al., 2017). These observations suggest that mere exposure to permissive cells and host CD81 or SR‐B1 is insufficient to render sporozoites immediately competent for productive invasion. Conversely, studies with *P. berghei* have shown that an impairment in productive invasion, either through disruption of the P36 or P52 encoding genes (Ishino et al., 2005b) or upon blockage of host CD81 or SR‐B1 (Manzoni et al., 2017) results in a significant increase in CT activity. While these results might suggest that sporozoite 6‐cys proteins and hepatocyte CD81 and SR‐B1 could play a direct role in parasite switching from CT to productive invasion, they could also imply that sporozoites that cannot productively invade simply continue to traverse through cells.

On the parasite side, the sporozoite 6‐cys protein P36 is proposed to be another key determinant of sporozoite sensing of the host for productive invasion (Manzoni et al., 2017). As stated before, it is plausible to consider that P36 and CD81/SR‐B1 are involved downstream of sporozoite activation and trigger rhoptry secretion and formation of the MJ for productive invasion. This role would be similar to the function of the merozoite micronemal protein EBA175 in *P. falciparum*, which binds to its erythrocyte receptor glycophorin A and triggers rhoptry discharge (Singh et al., 2010).

Following productive invasion, sporozoites must inactivate their CT machinery in order to avoid rupturing the PV membrane. This could be achieved, at least in part, by a pH‐sensing mechanism and the nonfusogenic nature of the PV, which avoids acidification by host cell lysosomes (Risco‐Castillo et al., 2015). In contrast, fusion of TVs with the host cell lysosomes may lead to their acidification, which in turn could activate PLP‐1 for sporozoite egress (Risco‐Castillo et al., 2015). The role of host cell lysosomes is probably more complex, as a recent study showed that *P. yoelii* sporozoites induce exocytosis of host cell lysosomes, which may promote parasite entry (Vijayan et al., 2019). In addition to pH, other mechanisms may regulate the activity of sporozoite PLP‐1, at the level of microneme secretion, membrane binding, or protein oligomerization for pore formation.

## 5 MICRONEMES ARE KEY SECRETORY ORGANELLES FOR SPOROZOITE CELL TRAVERSAL AND PRODUCTIVE INVASION

**FIGURE 2:**
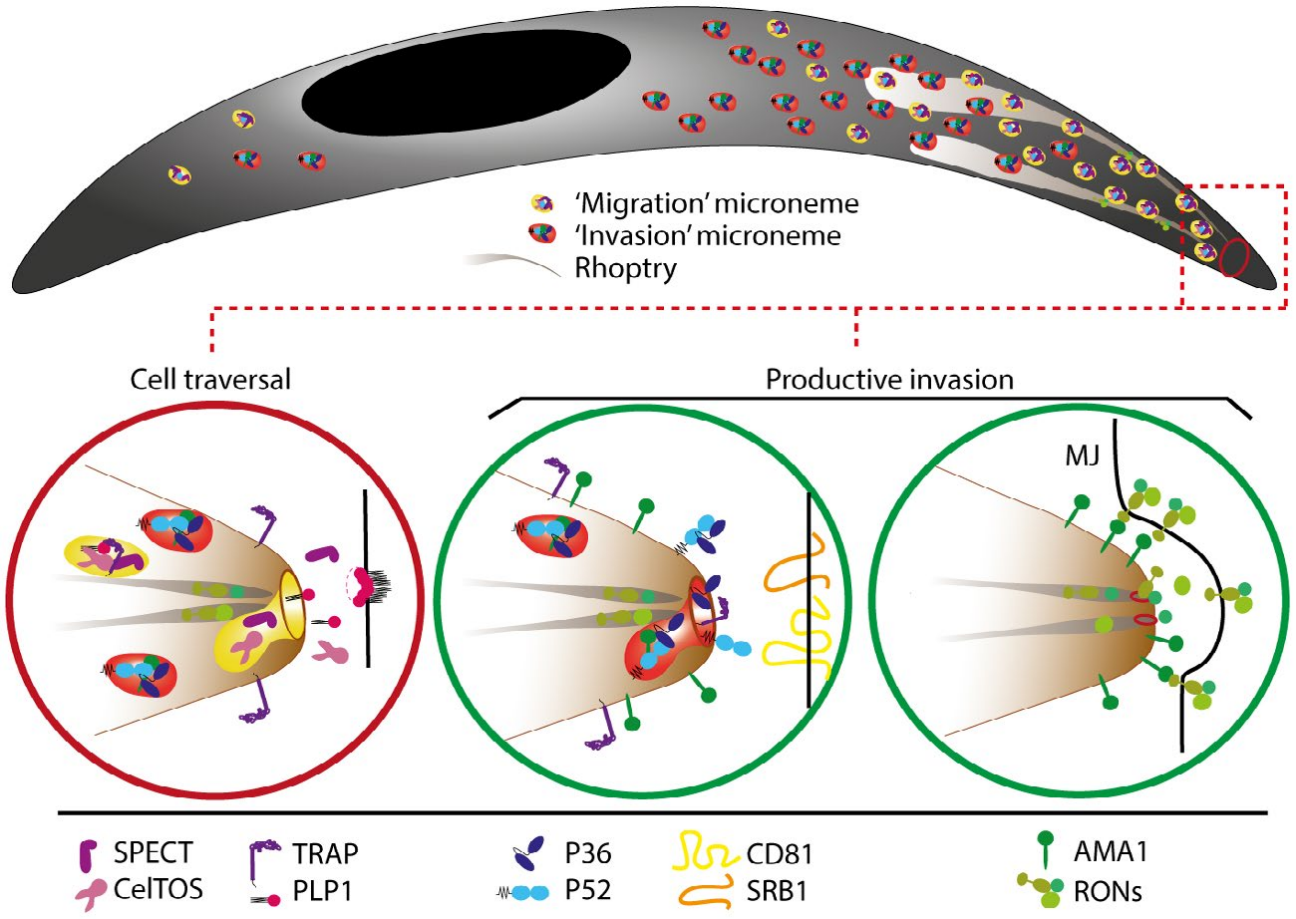
Hypothetical model for controlled secretion of distinct microneme subsets during sporozoite cell traversal and productive invasion. The functional and temporal uncoupling of cell traversal and productive invasion supports a model where sporozoite micronemes consist in discrete subpopulations that are temporally secreted and fulfill distinct functions. During cell traversal (red circle, left), secretion of migration‐specific micronemes would release proteins involved in gliding motility (e.g., TRAP) and membrane breaching (e.g., SPECT, PLP‐1, and CelTOS). Sporozoite activation may subsequently trigger the secretion of micronemes containing proteins essential for commitment to productive invasion (green circle, middle), including 6‐cys proteins (P36 and P52). Direct or indirect interaction of 6‐cys proteins with host receptors (CD81, SR‐B1) would then trigger the discharge of the RON proteins from the neck of the rhoptries, followed by export of the RON complex into the host cell and assembly of the moving junction (MJ), putatively through interaction of RON2 with parasite AMA1 (green circle, right).

Microneme secretion is a central process underpinning sporozoite migration and host cell invasion. Micronemes are small secretory vesicles present in the apical complex of all invasive stages of apicomplexan parasites (reviewed in Dubois & Soldati‐Favre, 2019). *Plasmodium* sporozoites harbor numerous micronemes, in contrast to merozoites that have relatively few. After fusing with the sporozoite plasma membrane, micronemes release sporozoite micronemal proteins (hereafter referred to as SpMICs) onto the parasite surface and/or into the extracellular milieu, sometimes after proteolytic cleavage. SpMICs form a diverse group of proteins with various functions in sporozoite motility (e.g., TRAP), traversal (e.g., SPECT‐1, PLP‐1), or productive invasion (e.g., P36, P52). How different SpMICs fulfill specific functions at distinct locations and times is currently enigmatic. One possibility is that the activity of secreted SpMICs varies depending on the cellular context and/or environmental cues, such as the pH, which regulates PLP‐1 function (Risco‐Castillo et al., 2015). However, the functional and temporal uncoupling of CT and productive invasion also supports the alternative hypothesis that SpMICs are secreted from distinct subpopulations of micronemes (Figure 2). In such a model, SpMICs involved in sporozoite migration (such as PLP‐1) could be enriched in “migration‐specific” micronemes, whereas proteins involved in host cell invasion (such as P36) would be contained inside “invasion‐specific” micronemes. SpMICs associated with gliding motility (such as TRAP) may localize to both subsets, as motility is required for both migration and invasion (Figure 2). Spatial compartmentalization and temporal release of discrete subpopulations of micronemes may not only ensure successful invasion of final target cells in the liver, but could also contribute to evasion of the host immune response, by limiting the exposure of key parasite ligands, such as the 6‐cys proteins, to neutralizing antibodies. Interestingly, in parasites that are activated by temperature switch or incubation with serum albumin, certain micronemal proteins such as TRAP can be readily detected on the sporozoite surface, while others cannot (Carey et al., 2014; Swearingen et al., 2016), consistent with the idea that not all micronemes secrete the same components.

Heterogeneity in micronemes has been previously documented in *Toxoplasma* tachyzoites, based on differential trafficking and/or localization of independent micronemal proteins (Kremer et al., 2013; Matthiesen et al., 2001; Venugopal et al., 2017). In these parasites, at least two microneme populations can be distinguished, apical micronemes clustered around the conoid and peripheral/lateral micronemes distributed along the parasite body (Dubois & Soldati‐Favre, 2019). These two subsets differ in their composition and involve distinct trafficking pathways (Matthiesen et al., 2001; Morlon‐Guyot et al., 2018). Differential localization and/or secretion of the micronemal proteins AMA1 and EBA175 has also been reported in *P. falciparum* merozoites, consistent with the existence of distinct microneme subtypes also in *Plasmodium* (Absalon et al., 2018; Ebrahimzadeh et al., 2019; Healer et al., 2002).

Until recently, the subcellular distribution of SpMICs had not been assessed in individual sporozoites. In a recent study, Arredondo et al analyzed the distribution of P36, P52, and TRAP in *P. yoelii* sporozoites by immune‐electron microscopy after dual labeling, and showed the co‐presence of P36 and P52, P36 and TRAP, and P52 and TRAP within a subset of micronemes (Arredondo et al., 2018). This study supports the idea that micronemes are heterogenous since only one third of the micronemes contained both P36 and P52, and around 20% of the micronemes contained neither P36, P52 nor TRAP (Arredondo et al., 2018). Nearly half of the micronemes containing TRAP were also labeled for P52, consistent with the idea that TRAP may have additional functions beyond motility during productive host cell invasion, possibly for sporozoite internalization through the MJ or through host cell recognition (Dundas et al., 2018; Klug et al., 2020; Matuschewski et al., 2002).

Although the distribution of CT‐associated SpMICs relative to invasion‐related SpMICs among sporozoite micronemes is unknown, it can be hypothesized that the hierarchical exocytosis of “migration micronemes” followed by “invasion micronemes” underlies the functional switch from a migratory to an invasive mode (Figure 2). Such a model would also explain why CT‐deficient sporozoites, despite lacking individual SpMICs such as PLP‐1, only commit to productive invasion after a significant delay, similar to WT parasites (Risco‐Castillo et al., 2015). Nevertheless, whether distinct extrinsic and/or intrinsic stimuli and signaling cascades contribute to the secretion of microneme subsets yet remains to be determined.

## 6 SIGNAL TRANSDUCTION IN PLASMODIUM SPOROZOITES DURING MIGRATION AND INVASION

Studies in *Plasmodium* and *Toxoplasma* have shed light on key mediators and downstream effectors that trigger microneme secretion during egress, motility, and invasion (reviewed in Bisio & Soldati‐Favre, 2019). In particular, second messengers such as cyclic nucleotides and calcium (Ca^2+^) play a central role in these processes. Ca^2+^‐mediated regulation is known to play a prominent role in sporozoite gliding and invasion (Carey et al., 2014; Kebaier & Vanderberg, 2010; Ono et al., 2008). Upstream of Ca^2+^, cyclic guanosine monophosphate (cGMP) activates protein kinase G (PKG), resulting in the release of intracellular Ca^2+^ (Carey et al., 2014; Kebaier & Vanderberg, 2010; Ono et al., 2008), via the action of phosphoinositide phospholipase C (PI‐PLC) and the production of inositol triphosphate (IP3) (Carey et al., 2014). The use of PKG inhibitors and PKG mutagenesis showed that PKG is required in *P. berghei* for TRAP secretion, sporozoite gliding motility and host cell invasion (Govindasamy et al., 2016; Govindasamy et al., 2019; Panchal & Bhanot, 2010). Serum albumin, a natural agonist for PKG‐dependent microneme secretion (Brown et al., 2016), also activates Ca^2+^‐dependent motility in sporozoites (Kebaier & Vanderberg, 2010). Consistent with the role of cGMP, inhibitors of phosphodiesterases (PDE), which increase cytosolic cGMP by blocking PDE‐mediated hydrolysis of cyclic nucleotides, enhance sporozoite motility (Kebaier & Vanderberg, 2010). In particular, PDEγ is required in sporozoites to regulate the levels of cGMP for sporozoite motility and transmission to the mammalian host (Lakshmanan et al., 2015).

Downstream effectors of Ca^2+^ flux include several sporozoite calcium dependent protein kinases (CDPKs). CDPK4 conditional knockout mutants display a twofold decrease in productive invasion of HepG2 cells, and CDPK4 chemical inhibition leads to reduced sporozoite motility in *P. berghei* (Govindasamy et al., 2016). CDPK1 and CDPK5 also contribute to sporozoite gliding motility and host cell invasion, although increased cGMP can compensate for the functional loss of CDPK1 and CDPK5 during sporozoite invasion (Govindasamy & Bhanot, 2020). Disruption of CDPK6 gene in *P. berghei* caused an increase in sporozoite CT activity and reduced hepatocyte invasion, associated with a defect in CSP cleavage (Coppi et al., 2007). CDPK6 may participate in signal transduction resulting in secretion of the protease that cleaves CSP upon sporozoite contact with highly sulfated HSPGs (Coppi et al., 2007). In addition to CDPKs, another potential downstream effector is the Ca^2+^‐responsive phosphatase calcineurin, for which conditional depletion was also shown to reduce sporozoite invasion in *P. berghei* (Philip & Waters, 2015).

Signaling through cyclic adenosine monophosphate (cAMP) and protein kinase A (PKA) is essential for merozoite invasion in *P. falciparum* (Dawn et al., 2014; Patel et al., 2019; Wilde et al., 2019), where PKA‐dependent phosphorylation of AMA1 plays an important role (Leykauf et al., 2010). The cAMP signaling pathway is also thought to be important in *Plasmodium* sporozoites. Chemical inhibitors of adenylate cyclases (ACs) or PKA inhibit TRAP secretion, sporozoite motility and host cell invasion in *P. berghei* and *P. yoelii* (Kebaier & Vanderberg, 2010; Ono et al., 2008). Disruption of ACα gene in *P. berghei* resulted in a defect in TRAP secretion and sporozoite infectivity (Ono et al., 2008). Reciprocally, induction of cAMP by the AC activator forskolin was shown to enhance motility in *P. berghei* sporozoites (Kebaier & Vanderberg, 2010) and to induce TRAP exocytosis in *P. berghei*, *P. yoelii,* and *P. falciparum* sporozoites, in a K^+^‐dependent manner (Ono et al., 2008). In *P. falciparum* merozoites, a low K^+^ environment activates ACβ, leading to a rise in cAMP levels, activation of PKA and Ca^2+^‐dependent microneme secretion that are essential for erythrocyte invasion (Dawn et al., 2014). A similar pathway may operate in *Plasmodium* sporozoites, where a shift from low to high K+ is associated with decreased CT activity, possibly reflecting the downregulation of microneme secretion (Kumar et al., 2007). Using the Flp/FRT‐based conditional mutagenesis system (Carvalho et al., 2004), Choudhary et al reported that the catalytic subunit of PKA (PKAc) is not required for sporozoite motility and hepatocyte infection (Choudhary et al., 2019). However, incomplete PKA depletion due to protein carryover cannot be ruled out, as was observed for PKG using the same genetic system (Falae et al., 2010; Govindasamy et al., 2016).

In most of the aforementioned studies, interfering with signal transduction pathways involved in microneme secretion had major impacts on sporozoite gliding motility, which is necessary for CT and productive invasion. How signaling cascades integrate to control sporozoite microneme secretion in a timely and spatially controlled manner to induce a switch from traversal to productive invasion is, therefore, difficult to disentangle. In *T. gondii*, microneme secretion plays a key role in parasite egress after intracellular replication. Tachyzoite egress is followed by reinvasion of a host cell, which is associated with a new burst of microneme secretion associated with rhoptry discharge for the formation of the MJ (Bullen et al., 2016). Multiple rounds of microneme secretion are probably necessary in *Plasmodium* sporozoites during their journey from the skin to the liver. It is thus plausible that sequential exocytosis of discrete microneme populations during sporozoite migration or upon productive invasion may rely on successive waves of Ca^2+^ release, possibly from distinct stores. Distinct extrinsic stimuli may initiate successive cGMP and/or cAMP signals. Alternatively, distinct downstream effectors, including Ca^2+^‐dependent enzymes or proteins that target micronemes to the plasma membrane for exocytosis (Bullen et al., 2016; Dubois & Soldati‐Favre, 2019; Ebrahimzadeh et al., 2019), may selectively trigger secretion of “migration‐specific” or “invasion‐specific” microneme subsets.

## 7 CONCLUSION AND FUTURE DIRECTIONS

The *Plasmodium* sporozoite stage has been a leading target for the development of malaria prophylactic strategies. Our understanding of how sporozoites achieve their complex journey from the skin to the liver, to ultimately invade and replicate inside hepatocytes, has greatly improved, but major questions still remain unanswered. Questions pertaining to the molecular mechanisms of productive invasion, or how distinct micronemal effectors fulfill distinct functions at different places and times still remain open. Moreover, questions concerning the existence of specialized micronemal subsets and the regulation of their secretion or the signaling cascades controlling the switch from a migratory to an invasive state and if all these mechanisms are conserved across all *Plasmodium* species are yet unresolved.

Although recent technological advances in gene targeting strategies will definitely help to address some of these questions, novel robust methods to distinguish CT from productive invasion events are also needed to capture the elusive sporozoite junction and dissect the mechanisms underlying the formation of the PV. Malaria sporozoites have evolved highly efficient mechanisms relying on coordinated secretion of apical organelles to safely travel from the inoculation site in the skin to their final niche in the liver. A better understanding of these mechanisms may contribute to the identification of potential targets for novel antimalarial intervention strategies.
